# Delayed post-polypectomy perforation: A case report of a 65-year-old man at 54 days post-procedure

**DOI:** 10.1097/MD.0000000000045311

**Published:** 2025-11-14

**Authors:** Donghyoun Lee, Beom Jun Lee, Jung Woo Yi, Robert Kim, Yuchul Jeong

**Affiliations:** a Department of Surgery, Jeju National University Hospital, Jeju National University School of Medicine, Jeju-si, Jeju-do, Republic of Korea; b St. Mary’s Best ENT Clinic, Seoul, Republic of Korea; c Department of Radiology, Kangbuk Samsung Hospital, School of Medicine, Sungkyunkwan University, Seoul, Republic of Korea; d Department of Medical and Pharmaceutical Affairs, Doctor Consult, Seoul, Korea; e Department of Internal Medicine, Chungna Good Hospital, Incheon, Republic of Korea.

**Keywords:** colonoscopy, colorectal neoplasms, diagnosis, differential, polyps, tomography, X-ray computed

## Abstract

**Rationale:**

Post-polypectomy perforation (PPP) is an uncommon complication, usually occurring within 24 hours of colonoscopic polypectomy. Delayed perforation beyond several days is extremely rare.

**Patient concerns:**

A 65-year-old man underwent colonoscopic removal of a 6-mm sessile tubular adenoma in the descending colon using a hot snare.

**Diagnoses:**

Six hours later, he developed abdominal pain with computed tomography findings consistent with post-polypectomy electrocoagulation syndrome and improved after conservative management. Fifty-four days later, he re-presented with severe abdominal pain; computed tomography revealed free intraperitoneal air and colonic necrosis.

**Interventions:**

Emergency subtotal colectomy confirmed PPP.

**Outcomes:**

The patient recovered well and remained asymptomatic at 6-month follow-up.

**Lessons:**

This case highlights that PPP can occur several weeks after polypectomy and may initially mimic post-polypectomy electrocoagulation syndrome. Clinicians should remain alert to late complications in patients with recurrent or persistent abdominal symptoms after polypectomy.

## 1. Introduction

Colorectal cancer (CRC) is ranked as the third most common and the second most common cause of cancer death worldwide. Use of colonoscopy with polypectomy (CP) is the gold standard for screening and prevention of CRC.^[1,2]^

A popular diagnostic modality for CRC, a screening colonoscopy is sometimes combined with the removal of polyps, thus termed as a CP. It serves as a method for removing precancerous polyps from the colon.^[3]^ While a CP is usually safe, it may cause some potential complications, including bleeding and perforation, which are rare but serious.^[4]^ Of these, perforation, termed as post-polypectomy perforation (PPP), deserves special attention in that it can mimic intestinal perforation, but it does not always require surgery and shows a favorable prognosis.^[5]^ Its causes include mechanical forces against the bowel wall, barotrauma and direct result of therapy or tissue sampling.^[6]^ Its prevalence is significantly higher in association with therapeutic rather than diagnostic colonoscopy. Most cases of PPP occur within 24 hours post-procedure. Although less common, however, it may also occur at 2 to 3 days post-procedure.^[7]^

We experienced an extremely rare case of a 65-year-old man presenting with PPP at 54 days post-procedure. To our knowledge, this is not seen in a prior publication. Here, we report our case with a review of the literature.

## 2. Case presentation

**Figure 1.**
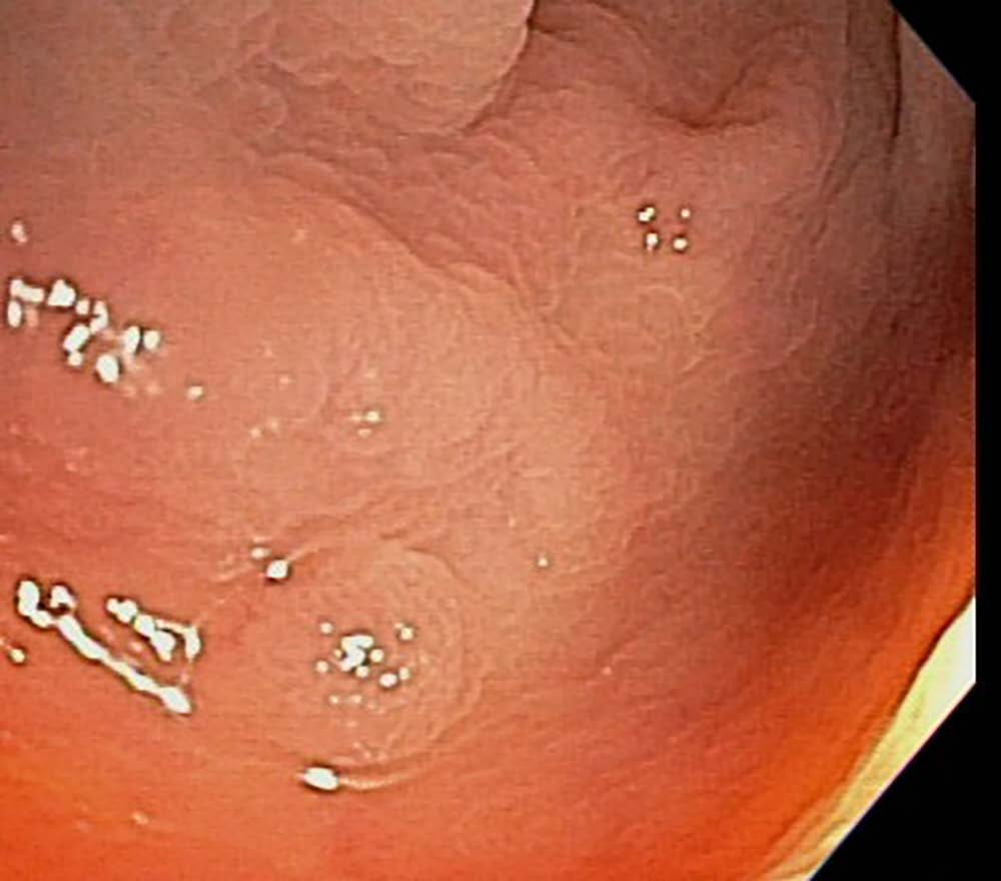
Colonoscopy showing a 6-mm sessile polyp in the descending colon, resected by hot snare polypectomy; pathology confirmed tubular adenoma.

**Figure 2.**
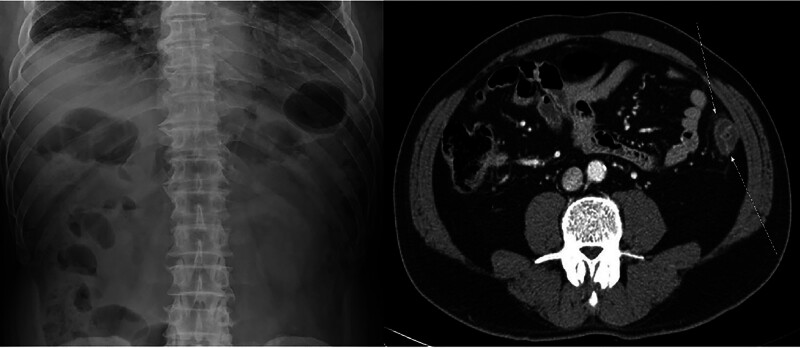
(Left) Plain abdominal radiograph without free air. (Right) Contrast-enhanced computed tomography demonstrating focal circumferential wall thickening of the descending colon (25 mm) without pneumoperitoneum, consistent with post-polypectomy electrocoagulation syndrome.

A 65-year-old man visited us to receive a regular CRC screening. On colonoscopy using pit and narrow-band imaging, a 6-mm sessile polyp was identified in the descending colon (Fig. 1). Pathology confirmed a tubular adenoma. Therefore, the patient was determined to undergo polypectomy. The lesion was lifted with a submucosal injection of epinephrine, methylene blue, and saline, and resected using a hot snare (standard oval snare, 40 W for 2–3 s). After the procedure, the patient complained of an approximately 6-hour history of acute isolated abdominal pain in the left lower quadrant. In the absence of fever, however, the patient had stable vital signs. On serum biochemistry, the patient had leukocytosis of 18.0 × 10^9^/L with 87% granulocytes. Moreover, chemical levels and liver function were within the normal limits. On X-rays, however, there was no free gas. The patient underwent intravenous contrast-enhanced computed tomography (CT) of the abdomen. This revealed the presence of a focal segment of the circumferential wall thickened from the descending colon, with maximum thickness of 25 mm on the lateral wall without evidence of pneumoperitoneum (Fig. 2). But the patient had a lack of signs of intestinal perforation. Therefore, in our case, a diagnosis of post-polypectomy electrocoagulation syndrome (PPES) was initially made.

**Figure 3.**
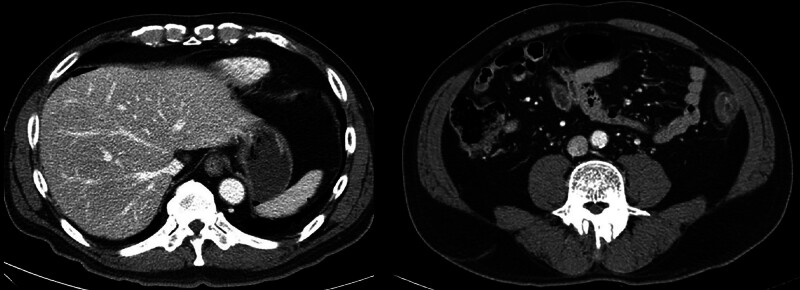
Abdominal computed tomography on day 54 showing free intraperitoneal air around the liver and extensive colonic necrosis, indicating delayed post-polypectomy perforation.

**Figure 4.**
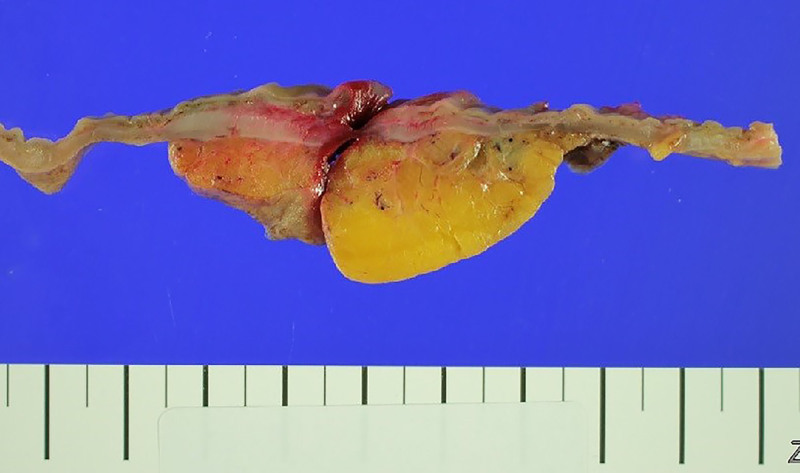
Gross intraoperative specimen of the descending colon showing perforation site covered by thick, yellow fibrinous exudate.

The patient received conservative treatments, such as a bowel rest, parenteral fluids and antibiotics (*e.g.*, the third-generation of cephalosporin), thus achieving a complete recovery of the abdominal pain and leukocytosis over the following 4 days. The patient was discharged on day 4. On day 54, the patient presented to the emergency department of our hospital with chief complaints of severe abdominal pain of 10/10 intensity on the visual analogue scale. On abdominal CT scans, the patient had air around the liver. This is suggestive of bowel perforation with extensive colon necrosis (Fig. 3). Moreover, there was an impression that the perforation site was located at the descending colon where the PPES was identified. Therefore, the patient underwent emergency open subtotal colectomy involving the terminal ileum, cecum, transverse, ascending and descending colon. This revealed that the perforation site was present at the descending colon. Grossly, the serosal surface showed thick yellow exudate at the perforation site (Fig. 4). Histopathology revealed acute serositis and lymphoid follicular hyperplasia without evidence of vascular thrombosis, ischemia, or mucosa-associated lymphoid tissue involvement.

Based on the above findings, the patient was finally diagnosed with PPP. Postoperatively, the patient recovered uneventfully, who was discharged on postoperative day 12 and remained well at a 6-month follow-up. The timeline of the patient’s clinical course is summarized in Table 1.

**Table 1:** The time line of the patient’s clinical course.

Time points	Key findings
Day 0	Colonoscopy and polypectomy
Day 0 (6 h)	Abdominal pain
Day 0–4	Hospitalization, CT, PPES diagnosis, conservative treatment
Day 4	Discharge
Day 54	Severe abdominal pain → CT → emergency subtotal colectomy
Postoperative period	Recovery/outcome

CT = computed tomography, PPES = post-polypectomy electrocoagulation syndrome.

## 3. Discussion

Colorectal polyps (CRP) are common benign lesions of the gastrointestinal tract and affect patients of all ages.^[8]^ Most adult cases of CRP occur due to family or personal history of CRC and are identified on colonoscopy for early CRC screening.^[9]^

A colonoscopy is widely accepted as the optimal modality for CRC screening and surveillance. It is generally a safe, well tolerated method; rates of its complications and those of CP are estimated at 0.3% and 2.3%, respectively. Complications of these 2 modalities include bleeding, perforation and PPES.^[10]^

PPP is a rare but serious complication of colonoscopy. Most cases of it occur immediately or within 48 hours of the procedure. Delayed perforations are exceptional; a few reports describe cases up to 5 to 7 days post-polypectomy, but none beyond 30 days were identified according to a review of the literature.^[11–13]^ Therefore, our case appears to be the first reported one of PPP at 54 days.

Possible mechanisms include: a contained microperforation sealed by omentum and peri-colonic fat, progressive necrosis of the coagulated bowel wall leading to late transmural defect and chronic low-grade inflammation and scarring culminating in eventual breakdown.^[4,14–16]^

As shown in the current case, it would be mandatory to make a differential diagnosis between the 2 entities. A CT scan of the abdomen and pelvis is is useful in making a differential diagnosis of PPP from PPES. In other words, a CT scan shows a focal thickening of the colonic wall with adjacent fat stranding, but no extramural air in a patient with PPES.^[12]^

We report the first case of PPP presenting 54 days after colonoscopic polypectomy. This case highlights the need for awareness of delayed perforation as a potential complication, the importance of differentiating PPP from PPES, and the necessity of careful follow-up in patients with post-polypectomy abdominal symptoms.

## Acknowledgments

The authors greatly thank Science Direct Inc. (https://www.sciencedirect.co.kr) for paying the article processing charge for the current article.

## Author contributions

**Conceptualization:** Yuchul Jeong.

**Data curation:** Beom Jun Lee.

**Formal analysis:** Beom Jun Lee.

**Funding acquisition:** Beom Jun Lee.

**Investigation:** Beom Jun Lee.

**Methodology:** Beom Jun Lee.

**Project administration:** Yuchul Jeong.

**Resources:** Jung Woo Yi.

**Software:** Jung Woo Yi.

**Supervision:** Yuchul Jeong.

**Validation:** Jung Woo Yi.

**Visualization:** Jung Woo Yi.

**Writing – original draft:** Donghyoun Lee, Robert Kim, Yuchul Jeong.

**Writing – review & editing:** Donghyoun Lee, Robert Kim, Yuchul Jeong.
